# Living with angina: rethinking angina management in ANOCA through lived experience

**DOI:** 10.3389/fcvm.2026.1956191

**Published:** 2026-10-05

**Authors:** Sarena La, Sarah Brown, Cindy McCall, Annette Pompa, Rosanna Tavella, Sivabaskari Pasupathy, John F. Beltrame

**Affiliations:** 1 School of Medicine, College of Health, Adelaide University, Adelaide, SA, Australia; 2 Department of Cardiology, Central Adelaide Local Health Network, Adelaide, SA, Australia; 3 Basil Hetzel Institute for Translational Health Research, Adelaide, SA, Australia; 4 International Heart Spasms Alliance, London, United Kingdom; 5 International Heart Spasms Alliance, Gold Coast, QLD, Australia; 6 International Heart Spasms Alliance, Catasauqua, PA, United States

**Keywords:** angina management, ANOCA, lived experience, patient advocacy, patient-centred care

## Abstract

Angina with non-obstructive coronary arteries (ANOCA) is an important cause of chronic angina and reduced quality of life. Despite advances in diagnostic testing and growing recognition of coronary vasomotor disorders, many of those living with ANOCA continue to experience ongoing symptoms despite treatment. The existing literature largely focuses on diagnostic methods and pharmacological management, while comparatively little attention has been paid to the lived experience of symptom control. The objective of this perspective paper, produced with the co-founders of the International Heart Spasms Alliance, is to explore angina management in ANOCA through the lens of lived experience and patient advocacy. Key challenges include the substantial impact of chronic angina on daily life, difficulties identifying effective therapies, the need to adapt to a new normal, reliance on self-management strategies, uncertainty surrounding acute symptom escalation, and the importance of support and validation. Improved angina management in ANOCA requires a holistic and individualised approach that combines mechanism-based treatment, multidisciplinary support, and meaningful incorporation of lived perspectives into clinical care and research. Successful angina management should be measured not only by symptom reduction, but also by improvements in quality of life and overall wellbeing.

## 1 Introduction

Chronic angina remains one of the greatest challenges faced by people living with angina with non-obstructive coronary arteries (ANOCA) (1). ANOCA encompasses heterogenous underlying mechanisms, including coronary vasomotor disorders. Despite advances in diagnostic testing and increasing recognition of these underlying mechanisms, many patients continue to experience recurrent symptoms that substantially affect quality of life (2). While substantial attention has been given to disease mechanisms (3, 4), diagnosis (5, 6), and pharmacological management (7, 8), the lived experience of managing chronic angina and its broader impact on daily life have been less extensively and systematically studied.

This perspective was co-produced with Cindy McCall, Sarah Brown, and Annette Pompa, lived experience partners and co-founders of the International Heart Spasms Alliance (IHSA), an international patient-led organisation supporting more than 6,000 people living with coronary vasomotor disorders. Cindy McCall, based in Australia, lives with Vasospastic Angina; Sarah Brown, based in the United Kingdom, also with Vasospastic Angina; and Annette Pompa, based in the United States, with Coronary Microvascular Dysfunction. Collectively, they bring decades of lived experience across different healthcare settings, together with sustained engagement with a broader international patient community through advocacy, peer support, educational initiatives, and ongoing dialogue. Drawing on these personal and collective experiences, this perspective explores the realities of angina management in ANOCA and opportunities to improve patient-centred care.

## 2 Discussion

As part of the manuscript co-production and authorship process, the three patient IHSA co-authors contributed their experiences and perspectives on living with ANOCA, including angina management strategies, interactions with healthcare providers, and unmet support needs. Their contributions together with insights from their ongoing engagement with the international ANOCA community, shaped the key themes explored below. These were further informed by preliminary findings from an ongoing international survey program called *Your Heart Speaks* (9), involving more than 1,000 people living with ANOCA, which captures lived experiences across diagnosis, symptom burden, treatment, and daily life. Together, these perspectives provide insight into the realities of living with and managing chronic angina, and opportunities to improve care.

### 2.1 Living with angina

For many people living with ANOCA, angina is not merely a symptom that occurs in isolation but a debilitating condition that impacts everyday life; it can be physically, emotionally, and socially demanding (10, 11). It can affect how they work, exercise, travel, socialise, and engage with family responsibilities. Those with ANOCA frequently describe having to modify daily routines, pace activities, or carefully plan to accommodate their symptoms. Preliminary data from the *Your Heart Speaks* survey program suggest that up to 40% of those with ANOCA stop working altogether (12), highlighting the substantial impact chronic angina can have on occupational functioning and financial security. Some restrict exercise despite understanding its health benefits, while others decline social engagements or alter travel plans because of concerns that their angina may worsen. Consequently, many describe a continual balancing act between maintaining participation in valued activities and avoiding symptom exacerbation.

Over time, living with angina may alter how individuals view themselves and their future. Those living with ANOCA who were previously active and independent often describe adjusting expectations, redefining personal goals, and learning to live within new limitations. As Cindy McCall reflected, “*I used to run on full steam, but now it's half steam, and that is okay. I am listening to my body and understanding what I need. This is my new normal.”* As advocates with decades of lived experience, the IHSA co-authors described a gradual process of acceptance—not as surrender to their condition, but as a shift in perspective. This journey is rarely straightforward and may involve periods of frustration, grief, and uncertainty as individuals come to terms with the ongoing impact of symptoms. Yet through experience, they have learned to prioritise what remains possible, adapting their lives in ways that support ongoing participation, purpose, and quality of life. In this context, acceptance becomes less about giving in to the condition and more about moving forward despite it.

### 2.2 Managing angina

#### 2.2.1 Recognising patterns and triggers

The IHSA co-authors emphasise the importance of understanding individual symptom patterns and triggers as part of developing an effective self-management strategy. While symptoms can remain unpredictable and recognising potential triggers does not prevent all episodes, developing an awareness of factors that influence the onset or exacerbation of angina symptoms may help individuals feel more in control of their condition. Symptom characteristics vary between individuals (1), with commonly recognised triggers including physical overexertion, emotional stress, lack of sleep, illness, temperature, and hormonal fluctuations. As Cindy McCall explained, “*Understanding your triggers is extremely important. It can help you plan accordingly.”* For example, Cindy McCall routinely carries warm clothing with her, even on hot Australian days, because exposure to cold environments, such as shopping centres, can provoke symptoms. Through their lived experience and advocacy work, the IHSA co-authors have observed that recognising symptom patterns often develops gradually over time. Beyond identifying specific triggers, they highlighted the importance of learning personal limitations, recognising changes in symptom patterns, and developing confidence in symptom interpretation. As Annette Pompa reflected, “*It is important to be proactive as a patient.”* In this context, understanding one's angina becomes a form of self-advocacy, enabling individuals to make informed decisions about daily activities and participate more actively in their own care. Although there is currently no standardised clinical approach to help patients systematically identify and track symptom triggers, clinicians may support this process by encouraging symptom tracking and facilitating discussions regarding individual symptom patterns and triggers.

#### 2.2.2 Navigating treatment by trial and error

Identifying treatments that provide meaningful symptom relief remains one of the greatest challenges in ANOCA care (7). Unlike many cardiovascular conditions, where evidence-based treatment pathways are well established, symptom management in ANOCA often involves a prolonged process of trial and error. While patients can achieve substantial improvements in symptoms, and some become largely or completely symptom-free following appropriate diagnosis and treatment, this is not the reality for all. Within the IHSA community, patients describe a journey of sequential medication trials, dose adjustments, and combination therapies in pursuit of symptom control. This process can be frustrating and emotionally taxing, particularly when symptoms remain uncontrolled despite adherence to treatment recommendations. Furthermore, treatment response is highly individualised; medications that provide substantial benefit for one patient may have limited efficacy in another, even among individuals with the same clinical diagnoses. As Annette Pompa explained, “*We are not static beings; we change. What helps you one year might not help you the next. The trial and error can be ongoing.”* Symptoms may evolve over time, treatment effectiveness may fluctuate, and what works at one stage of the disease may no longer be effective later.

The emergence of invasive functional angiography testing and stratified endotype-directed treatment strategies offers an important opportunity to improve symptom management by targeting the underlying mechanisms responsible for symptoms (5). Nevertheless, even with mechanism-guided treatment, some patients continue to experience residual symptoms or develop refractory angina, defined as chronic angina (>6 months) despite maximally tolerated conventional anti-anginal therapy (8). While the prevalence of refractory angina in ANOCA remains unknown, these patients are often those with the greatest symptom burden and represent an important area of unmet clinical need (8). Consequently, successful treatment is not always defined by complete elimination of pain, but rather by achieving acceptable symptom control, improving quality of life, and restoring participation in meaningful activities. Establishing realistic treatment expectations, engaging in shared decision-making, and recognising the need for ongoing treatment adjustment are therefore important components of patient-centred care. Given the heterogeneity of ANOCA, effective angina management necessitates a personalised care approach that is tailored to both the underlying disease mechanism and the individual patient's experiences, preferences, and treatment goals.

#### 2.2.3 Strategies beyond pharmacological therapy

For many patients, angina management extends far beyond pharmacological therapy. While medications remain an important component of care, patients frequently describe developing a personalised toolkit of strategies to help manage symptoms, maintain wellbeing, and navigate the emotional burden associated with angina. These approaches are not intended to replace medical treatment but rather complement it, particularly when symptoms remain only partially controlled by medical therapy. As Sarah Brown observed, “*Pain is personal. It is subjective. It belongs to the person living with the pain.”* This perspective was reflected in the diverse approaches those living with ANOCA use to manage symptoms. The IHSA co-authors highlight the value of practical coping strategies that enable them to continue participating in everyday life despite experiencing symptoms. Commonly reported strategies included activity pacing, planning for anticipated triggers, relaxation techniques, breathing exercises, mindfulness, meditation, and maintaining physical activity within individual limits. Some members of the IHSA community have also described using transcutaneous electrical nerve stimulation (TENS) to help manage angina episodes. Although neurostimulation has been investigated in refractory angina (13), evidence supporting TENS specifically for symptom management in ANOCA is limited, and further research is required. Other specialised non-pharmacological and device-based therapies have also been explored for refractory angina, although their use and supporting evidence in ANOCA remain limited (8). It is emphasised that remaining calm during symptom episodes is important, as panic may amplify distress even when it is not the underlying cause of symptoms. Others described prioritising sleep, maintaining routines, and focusing on activities that promoted overall wellbeing. Participation in cardiac rehabilitation may also provide education, support, and reassurance; however, many report that existing programmes are not specifically tailored to the challenges associated with ANOCA. Collectively, these experiences highlight that effective angina management requires more than symptom reduction alone and should address the broader physical, emotional, and behavioural aspects of living with angina.

### 2.3 Navigating acute episodes

Many patients describe the challenge of distinguishing their usual angina symptoms from unstable symptoms that may warrant urgent medical assessment. Although recurrent episodes may increase familiarity with symptom patterns, deciding when to seek medical attention can remain difficult, particularly when symptoms vary in intensity, duration, or presentation. These decisions are often influenced by previous healthcare experiences. Some individuals report reluctance to attend emergency departments because their symptoms have previously been dismissed or attributed to anxiety, while others describe concerns about overlooking a potentially serious cardiac event. Consequently, acute symptom escalation can be accompanied by considerable uncertainty and emotional distress. Personalised management plans may help address these challenges by providing clear guidance regarding symptom recognition, medication use, escalation strategies, and indications for urgent assessment.

Healthcare professionals should recognise that people living with ANOCA may experience genuine and distressing cardiac symptoms despite the absence of obstructive coronary disease. Compassionate, patient-centred care begins with acknowledging that the patient's symptoms are real and should not be dismissed. Acknowledging previous diagnoses, validating symptom experiences, and ensuring continuity of care can improve patient experiences and healthcare engagement. Individualised care plans, developed collaboratively with patients and communicated across healthcare settings, are widely recognised as an important component of chronic disease management. In ANOCA, they may provide greater confidence during acute episodes by outlining symptom recognition, medication use, escalation strategies, and indications for urgent assessment. Improved integration between specialised services, primary care, emergency departments, and allied health professionals may further support coordinated, patient-centred care and help patients navigate acute symptom episodes with greater confidence.

### 2.4 Support and connection

Chronic angina can be an isolating experience, particularly for individuals living with a condition that remains poorly understood within healthcare and the wider community. This lack of understanding can make it difficult for people to explain their condition to others, contributing to feelings of invalidation, loneliness, and social isolation. Within the IHSA community, the importance of feeling understood, believed, and supported is commonly emphasised by people living with ANOCA. When Sarah Brown first experienced symptoms, she explained, “*I had never met anyone else with the same condition. It was very isolating. I was very lonely.”* For many, the absence of connection with others living with similar conditions contributed to feelings of uncertainty and isolation, particularly during the diagnostic journey. Support from family members, friends, healthcare professionals, and peer networks was repeatedly identified as an important component of living well with ANOCA. However, these conversations are not always easy. Many describe how, over time, they became reluctant to discuss symptoms with family because they did not want to cause fear or become a burden. Others described loved ones struggling to understand the ongoing impact of recurrent angina. Patient advocacy and support organisations can therefore play an important role by providing validation, practical advice, education, and a sense of community. The International Heart Spasms Alliance was established in response to this need, creating opportunities for people to connect, share experiences, and advocate for improved awareness and care. As Sarah Brown reflected, “*We want to make life better for the people.”* These experiences suggest that support and connection should be viewed not as optional extras but as valuable adjuncts to care.

### 2.5 Opportunities to improve angina management

Those living with ANOCA consistently advocate for a more personalised, holistic, and patient-centred approach to angina management. Central to this vision is recognition that angina control remains a primary treatment goal, even when mortality risk may be comparatively low. Effective angina management begins with validation. Despite increasing awareness of coronary vasomotor disorders, many continue to report experiences of diagnostic uncertainty and symptom dismissal. From our preliminary *Your Heart Speaks* survey program data, three in four participants were told that their symptoms are non-cardiac, most commonly being attributed to anxiety (9). As Annette Pompa stated, “*To think that our condition is still being dismissed as non-cardiac chest pain or anxiety—it shouldn't be happening.”* Such experiences may contribute to delays in diagnosis, reduced trust in healthcare interactions, and feelings of invalidation.

Future models of care should incorporate mechanism-based diagnosis, individualised treatment plans, multidisciplinary support, improved clinician awareness, and greater emphasis on patient-reported outcomes and quality-of-life measures (7, 14) (Figure 1). Co-designed, personalised care plans that recognise patients as partners in their care may further improve self-management, continuity of care, and confidence when navigating both routine and acute symptom episodes. Research priorities should extend beyond disease mechanisms to include symptom burden, treatment satisfaction, self-management strategies, and outcomes that matter most to patients. Therefore, future models of research should incorporate people living with ANOCA to ensure that studies address issues most relevant to them. Ultimately, improving angina management requires a shift away from focusing solely on disease processes and towards understanding the lived experience of chronic angina.

**Figure 1:**
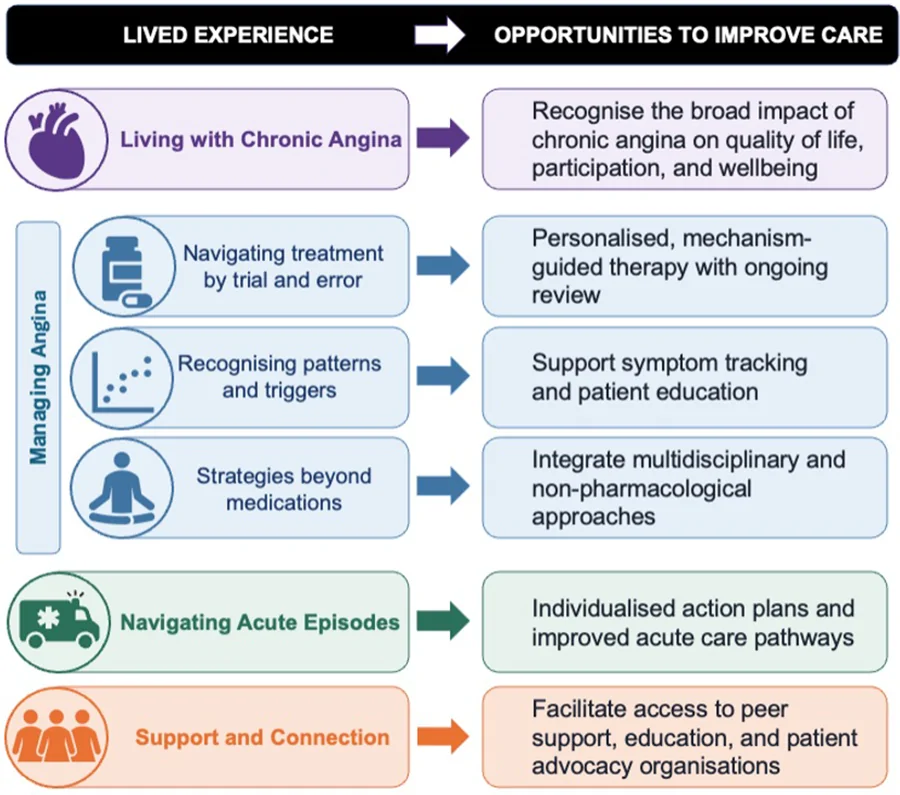
Key themes and opportunities for improving angina management in ANOCA.

## 3 Conclusion

Chronic angina remains one of the greatest challenges faced by people living with ANOCA. Although advances in diagnosis and mechanism-directed treatment have improved understanding of the condition, many patients continue to experience significant symptom burden. Effective angina management extends beyond medication selection and includes validation, self-management support, emotional wellbeing, and clear pathways for managing acute symptoms. Incorporating patient perspectives into clinical care, service design, and research offers an important opportunity to improve outcomes and quality of life for individuals living with ANOCA.

## Data Availability

The original contributions presented in the study are included in the article/Supplementary Material, further inquiries can be directed to the corresponding author.
